# Designing a Biomaterial Approach to Control the Adaptive Response to a Skin Injury

**DOI:** 10.3390/ma15186366

**Published:** 2022-09-13

**Authors:** Dale Feldman

**Affiliations:** Department of Biomedical Engineering, University of Alabama at Birmingham, Birmingham, AL 35294, USA; dfeldman@uab.edu; Tel.: +1-(205)-807-2445

**Keywords:** wound healing, adaptive response control, burn treatment, pressure ulcer treatment

## Abstract

The goal of this review is to explain how to design a biomaterial approach to control the adaptive response to injury, with an emphasis on skin wounds. The strategies will be selected based on whether they have a reasonable probability of meeting the desired clinical outcome vs. just comparing the pros and cons of different strategies. To do this, the review will look at the normal adaptive response in adults and why it does not meet the desired clinical outcome in most cases. In addition, the adaptive response will be looked at in cases where it does meet the clinical performance requirements including animals that regenerate and for fetal wound healing. This will lead to how biomaterials can be used to alter the overall adaptive response to allow it to meet the desired clinical outcome. The important message of the review is that you need to use the engineering design process, not the scientific method, to design a clinical treatment. Also, the clinical performance requirements are functional, not structural. The last section will give some specific examples of controlling the adaptive response for two skin injuries: burns and pressure ulcers. For burns, it will cover some preclinical studies used to justify a clinical study as well as discuss the results of a clinical study using this system. For pressure ulcers, it will cover some preclinical studies for two different approaches: electrical stimulation and degradable/regenerative scaffolds. For electrical stimulation, the results of a clinical study will be presented.

## 1. Introduction

This review will cover strategies to control the adaptive response to injury using biomaterials. The emphasis will be on skin wounds. In addition, strategies will be selected based on whether they have a reasonable probability of meeting the desired clinical outcome(s). This requires establishing a minimum acceptable level of clinical performance (both minimum clinical benefit and maximum clinical harms (side effects). Therefore, the purpose of this review is not just to compare the pros and cons of different strategies but also show how to select strategies that can meet the desired clinical outcome(s).

To do this the review will look at the normal adaptive response in adults and why it does not meet the desired clinical outcome(s) in most cases. In addition, the adaptive response will be looked at in cases where it does meet the clinical performance requirements including animals that regenerate and for fetal wound healing. This will lead to how biomaterials can be used to alter the overall adaptive response to allow it to meet the desired clinical outcome(s).

Normally, the initial adaptive response is inflammation. Then, it is the repair phase and then the remodeling phase. There are a number of things that can affect the timing and specific bioprocesses that occur due to injury. These include: the extent of injury, location of the injury, and amount of bleeding as well as the presence of foreign material(s), dead cells, infection, or an underlying pathology [1,2,3,4]. Although the strategies are similar for all types of tissue, the review will focus on soft tissue with specific examples related to the largest organ in the body: the skin. The review will start with the normal adaptive responses and why the response needs to be controlled. It will then cover general approaches and when and how biomaterials could be used. The next section will cover the process of selecting the specific biomaterial approach to control the adaptive response. The last section will give some examples of designing systems by using biomaterials to control the adaptive response for two skin injuries: pressure ulcers and burns.

For skin, there are three general strategies that include adding material to the wound: grafting, graft substitutes, and scaffolds. The first, grafting, is the “gold standard” and does not require the use of a synthetic biomaterial. However, tissue adhesives will be covered as a way to improve the clinical outcome for the “gold standard”.

Although the general approaches will be comprehensive, the specific approaches and examples will not be. This is because the approaches selected have to be capable of meeting the clinical performance requirements and the requirements are different for different clinical applications. For example, there are many different strategies to make skin graft substitutes, but based on current knowledge they do not meet the clinical performance design constraints for most applications due mostly to our inability to duplicate the structure and function at the scale of biomolecules [2,3,4,5]. In the future, however, this could change. Further, strategies that do not include a biomaterial will be covered to help understand their influence independent of a biomaterial and how close they are to the desired clinical performance requirements without an added biomaterial.

In order to show how to control the adaptive response, specific examples need to be given. There are many ways to control the adaptive response but the control must allow meeting the clinical performance requirements (which are currently not being met) or it is not useful [6]. In addition, each strategy has its own performance requirements in order to meet the clinical performance requirements. Too often researchers use the Scientific Method to show that using a different strategy makes a statistically significant improvement in control of the adaptive response, at a cellular or tissue level, and claim it is should be used clinically [6]. Better control, however, is only useful if it allows for meeting the clinical performance requirements that are not currently being met [6]. Being better at the cellular or tissue level only matters if it allows the treatment to meet the performance requirements that are necessary to meet the clinical performance requirements [6].

## 2. The Adaptive Response

One important component of considering something to be living is the ability to respond to stimuli. For this review, the response to stimuli will be called the adaptive response. The goal of this review is to look at ways to control this adaptive response to injury in general and with some specific examples related to skin injuries using biomaterials. This section will look at the normal response to injury (adaptive response to the stimulus of injury). It will also look at differences in this adaptive response between species as well as between adult and during fetal wound healing. An attempt will be made to explain the reasons for these differences in adaptive responses. Ultimately, a theory will be presented and tested by showing that successful control strategies are consistent with this theory. The intent is not to prove the theory, but to see if the theory is a useful way to help select effective strategies for controlling the adaptive response.

In essence, each strategy used is an additional stimulus, which leads to a modification of the overall adaptive response. The goal is to understand how additional stimuli can be used to modify the normal adaptive response to injury in order to achieve the desired outcome. Although the emphasis will be on the use of biomaterials as graft substitutes or scaffolds, in some cases, the adaptive response to stimuli without the use of a biomaterial will be explored.

### 2.1. Adaptive Responses to Injury

There are three main types of adaptive responses to injury. In humans, all three occur and the relative amount of each, for a given injury, is dependent on a number of things including location, age, the extent of injury, and treatment. Two lead to recovery of structure: regeneration and hypertrophy (structures grow larger to fill the space). The third response is a repair process where contraction and scarring are used to reconnect the wound edges [1,2,7,8]. Clinically, the adaptive response is observed at the organ level, but it is the cellular level events (bioprocesses) that determine the adaptive response [7,8,9].

### 2.2. Regeneration

In adult humans, the adaptive response to injury is different among the four tissue types (epithelium, muscle, nerve, and connective tissue), with epithelium being the only one that heals predominantly by regeneration with little scarring [2,7]. The regenerative ability of the epithelium, however, is limited by cellular mitosis and migration rates and requires assistance, in some cases, to reduce scarring in underlying layers.

The other three types have some regenerative ability, typically in small size wounds. Regeneration is limited by the rate of angiogenesis (getting blood supply to the area) as well as the speed of re-establishing the epithelial layer. For skin grafts, the blood supply from the wound edges (including the wound bed) must attach to the vessels in the graft and it still takes a few weeks to have relatively normal blood flow in the graft [10]. Graft substitutes currently require blood vessels to grow in from the edges, extending the time to “heal-in” and hampering our ability to create actual graft substitutes [2,5,10,11,12,13,14,15,16,17,18,19]. It also makes the seeding of scaffolds *in vivo* an issue if the blood supply cannot reach the cells before they die [2,5,10,11,12,17].

### 2.3. Graft Healing

To understand the adaptive response to skin injury, it is important to look at the adaptive response using the “gold standard”: skin grafts. Although there are different types of grafts, the review will focus on free autografts (the “gold standard” unless otherwise stated), since that is what we are trying to replace with a graft substitute.

Understanding how grafts heal in can help show why graft substitutes are not feasible yet (to achieve the desired clinical performance). This leaves scaffold and tissue adhesives as the two currently available strategies using biomaterials.

The key for skin grafts is the attachment of blood supply from the underlying bed to the graft—both speed and completion. Although the loss of blood to tissues or organs can cause irreparable damage in 5 h or less, grafts can take about a week to heal in without much damage [10], with blood vessel ingrowth probably in the mm/week range (some claim 5 μm per hour and therefore about 1 mm/week) and cells needing to be within 0.1 mm of the blood supply to survive. Any graft over a few mm thick will only survive if the underlying blood vessels reconnect and use the blood vessels of the graft [1,2,8,10]. This requires the graft to be securely attached to the bed to allow a significant amount of reconnection [10]. If the old vessels [10] are used they probably need to be re-epithelialized—especially near the epidermal surface. Split thickness grafts are often used because they meet this criterion (0.25 to 0.5 mm thick) [1,2,10].

For a free graft, it takes about a day for vessels from the wound bed to attach to vessels in the graft and about 5 days to complete the vascular tree (if it has one already), and then a few more days before flow actually begins [10]. If there are no blood vessels (or conduits) in the graft or the initial attachment is delayed, the graft may not survive [10]. This can be all or just parts of the graft that do not receive blood supply fast enough [10]. Therefore, making sure the graft is securely attached to a healthy wound bed is important [10].

Until we can solve the vascular problem, we cannot make tissue-engineered skin that heals in like a graft (graft substitute) [1,2,3,4,5,6,10]. At best, we can obtain a dermis first and then add the epithelial layer [5,20,21]. Again, cell seeding can also be an issue *in vivo*, if the blood supply cannot reach the cells fast enough for them to survive [22]. The cells that do not survive just serve as short-term drug delivery systems. With current technology, cell seeding *in vivo* should probably be limited to the outside of the wound or the outside portion of the scaffold (if they are to be incorporated into the scaffold as it heals) since the blood supply cannot reach the center of the wound fast enough [22].

### 2.4. Loss of Regeneration

Many species are able to regenerate large injuries via epimorphic regeneration [11,23,24,25], which appears to be lost as part of evolution since more complex animals are less likely to be able to do this [11]. Determining the evolutionary change should help in determining effective strategies for controlling the adaptive response. In humans, this ability also decreases as we age [11]. Fetal wound healing is regenerative with no visible scar [26]. An amputated digit can regenerate even in newborns, but the percentage distance from the fingertip that can grow back decreases over time [26]. This difference in regenerative ability as we age is not likely to be an evolutionary change, since the genes do not change over time. Understanding the changes that occur in fetal versus adult wound healing should also help in determining effective strategies for controlling the adaptive response. The goal would be to determine what additional stimuli need to be added to overcome the adaptive response changes due to evolution and aging.

Through evolution and natural selection, the primary adaptive response for humans has become scarring vs. regeneration [20]. Typically, this means the scarring rate occurs much faster than the regenerative healing rate [20]. In some cases, however, there can be mostly regenerative healing after injury (usually limited by the size of the defect) in tissue and organs including: connective tissue (blood and bone), epithelial tissue, smooth muscle, and the liver [3]. Additionally, almost all tissue types can be turned over (replacing older cells with newer ones) with the length of time dependent on the tissue location and function [1,2,7,8,9,11,27,28]. In addition, the rate of regenerative healing relative to scarring can be increased with exogenous growth factors or stem cells as well as electrical stimulation [1,2].

Normally, the mitotic rate (healing rate) of a tissue is related to its turnover rate [7,8]. For example, epithelium tissue has both the fastest healing rate and the quickest turnover rate [7]. Even in multilayer epithelium, such as the epidermis (7 layers), the basal cells can migrate over a wound at 2 mm/wk and it only takes 30 days for a basal cell to mature and move upwards (increasing its keratin concentration), helping to form the layers of the epidermis, which end up with a keratinized surface layer [1,3,7].

Therefore, it is not really a loss of regenerative ability, but a reduction in the regenerative healing rate vs. the scarring rate. Things that can alter the relative rates besides aging appear to be the healing time (related to the size of the wound), the amount of inflammation, and the ability to mobilize stem cells (which decreases with age and in certain disease states).

It is likely that the evolutionary change is related to time to heal. As the time to heal increases the relative amount of scarring vs. regeneration increases, but the ratio at any given healing time would be the evolutionary change [11,24,28]. Similarly, for epimorphic regeneration to occur, the epithelial layer must cover the wound within a certain time frame (which is species-dependent). There are, however, also individuals who are prone to scarring and can have hypertrophic scarring even for small wounds.

It appears that an increased scarring rate relative to regeneration rate is the evolutionary advantage vs. reducing the regenerative healing rate. As you move up the evolutionary tree, individual components tend to become larger and more complicated, requiring a longer time to heal. This would not require an evolutionary (genetic) change. However, changing the ratio of regenerative healing to scarring at a given time to heal would. This appears to be the case since some larger animals can still regenerate large parts of their body (including a lizard’s tail, an African Spiny Mouse’s skin, a rabbit’s ear, and for an axolotl salamander) [24]. It would also appear that other similar sized animals (that do not regenerate these parts) have had an increase in scarring relative to regeneration. For many of these animals, who can still regenerate large parts of their body, it serves as a defense mechanism [24,28]. A lizard losing its tail to distract its prey is a good example [24,28].

In most animals, however, it was probably an evolutionary disadvantage due to the time to heal and the metabolic demand [7,8,24]. The added healing time would make an animal easier prey as well as make the wound more susceptible to infection [24,27]. The increased metabolic demand would reduce their ability to reproduce [24,27]. This is probably why many mammals will kill any injured or deformed offspring [25].

Others have said that the evolutionary disadvantage comes from being warm-blooded and terrestrial [24,27]. Being warm-blooded would increase the risk of infection. Being terrestrial means that leg regeneration would be difficult due to the need for weight bearing during healing [24]. It also appears that the longer an animal can survive without food the more likely it is to regenerate, with larger and warm-blooded animals having a shorter survival time without food [24].

This all means that in adult humans, the normal adaptive response to injury is predominantly scarring vs. regeneration, with the evolutionary change being how quickly the scarring rate increases relative to the regenerative healing rate. In many cases, in today’s world, the evolutionary advantages of scarring vs. regeneration are not as important. Infection is not as big an issue as well as the need for quick healing; so now more regenerative healing would be the evolutionary advantage. However, it typically takes at least 50 generations for an evolutionary change to occur once the mutation starts showing up (which it has not [not counting science fiction movies]). Therefore, again there is a need to explore the use of additional stimuli to control the overall adaptive response. If in fact the bioprocess to control is time to complete healing, strategies to reduce time to heal need to be explored. Since normally there is little regenerative healing during the inflammatory phase of healing, good strategies could either shorten the inflammatory phase or speed healing in the repair phase.

#### 2.4.1. Mammalian Regenerative Healing

To develop these strategies, it is useful to determine why regenerative healing occurs in mammals (including fetal wound healing). There are only a few examples of epimorphic regeneration in adult animals; deer antlers and the ear of a rabbit [11]. Interestingly, other animals with similar ears (such as dogs) heal by scarring [11]. Again, most injured tissue in adult mammals heals predominantly by scarring and has to go through all three phases of the wound healing process (inflammation, repair, and remodeling). In some cases, usually small wounds, discontinuity or defects can heal with little to no scarring [11,23,24]. As previously mentioned, this can be by hypertrophy (skeletal muscle) or by organs that have limited regenerative ability (including bone, the liver, and epithelial tissue) [11]. Wounds that involve skin or penetrate the skin require epidermal closure for a blastema to form, which is not a requirement for damage to internal organs [11].

It is unlikely, however, that epimorphic regeneration will be a useful strategy in adult humans because all the requirements are difficult to recreate at the same time including: a skin injury that heals over quickly, has quick access to stem cells for the repair, has early enervation, and requires little to no inflammatory phase [8,11]. In addition, the size and complexity of adult human structures would require a long time to form and grow to adult size—it takes about twenty years for most human structures to reach adult size [28]. Again, although regeneration does occur, in some cases internally without a blastema, it is limited to small defects.

#### 2.4.2. Fetal Wound Healing

Again, understanding the difference between adult and fetal wound healing can help with determining strategies to control the adaptive response. Although there is no blastema, *in utero* healing is essentially scarless [28]. Although there are a number of biochemical differences between adult and fetal wound healing there are two factors that probably have the biggest effect: the availability of stem cells and the size of the defect [8,11,28]. Both affect the time to heal and the characteristics of the healed wound [2,4,8]. In a fetus, there is continual growth, with the presence of stem cells and progenitor cells playing a big part [2,4,8]. There is less of a need to recruit stem cells from blood and bone marrow [2,4,8]. This reduces the time needed to both start and complete the healing process. Whereas in adult wounds, little regenerative healing can happen during the inflammatory process, the locally available stem cells can start the regenerative process even during the inflammatory phase, in fetal wound healing. In adults, inflammation is required to clean up the wound from dead and necrotic tissue as well as debris; which are not normally present in fetal wounds. Inflammation also signals the repair cells to gather at the wound, which is also not necessary for fetal wounds. In addition, structures and defects in a fetus are smaller than in adults, leading to a shorter healing time.

As a fetus matures, although structures continue to become larger, the growth rate decreases. This would also slow the regenerative healing rate due, at least in part, to a reduction of available stem cells. *In utero*, the structures not only become larger, but more complex. After birth, the structures would continue to grow, with the rate decreasing over time. Both the reduction in stem cells (and growth rate) as well as the larger size of structures is probably why the percentage of a finger cut off still being able to regenerate gets smaller and smaller over time. It is, therefore, likely that there is no true scarless healing without epimorphic regeneration and the amount of scarring relative to regeneration increases as we age, since the time to heal increases due to a longer delay in regenerative healing, a decreased healing rate, and larger structures to heal [23,28].

### 2.5. Developing Strategies to Reduce Scarring and Increase Regeneration

With the evolutionary change, most likely being how the scarring rate increases relative to the regeneration rate as healing time increases and the main difference between fetal and adult wound healing is the time to heal, control strategies can be developed. Therefore, strategies should aim at reducing healing time by shortening the delay in healing during the inflammatory phase and/or increasing the healing rate in the repair phase.

This could be achieved by shortening the inflammatory phase by removing foreign material, necrotic tissue, etc. from the wound to reduce the amount of “cleaning up” that is necessary [2,4]. Additionally, to reduce or eliminate forces that could reinjure the wound, since most incision wounds can heal without a visible scar, even in adults, if the wound is clean and protected [2,4]. In addition, strategies to allow cells to handle the inflammatory response, as well as start regenerative healing early (including introducing stem cells), can shorten the delay in the regenerative healing normally seen. Additionally, strategies to speed the healing process in the repair phase will help to reduce the overall healing times, which could also include the introduction of stem cells.

Although how time to heal affects the amount of scarring is most likely the evolutionary change, it is important to understand what bioprocesses are responsible for this change in relation to the amount of scarring. It would be helpful to examine the normal adaptive response to injury to try to understand not only the specific evolutionary change but also where and when control strategies could be used effectively.

#### 2.5.1. The Normal Adult Adaptive Response

Since regenerative epimorphic healing does not occur in adults and hypertrophy only occurs in specific cases, healing of an injury typically goes through four overlapping phases: hemostasis, inflammation, repair, and remodeling. Hemostasis is stopping the bleeding (usually a fibrin clot) and is dependent on the type of wound as well as if there are any efforts to stop the bleeding (including suturing the wound).

Then comes the inflammatory phase, which is where the neutrophils and macrophages “clean up” any foreign material, dead tissue, and or products of hemostasis including: red blood cells, edema, and fibrin clot in order to prepare for the next phase [2,4]. Again, little regenerative healing occurs in adults; instead, there is the beginning of the repair phase with a well-vascularized provisional matrix and/or scarring to wall off any foreign material [2,11]. Additionally, if a healing wound is not sufficiently protected it can re-tear and start the inflammatory “clean up” process again.

The inflammatory phase transitions into the repair phase. In some cases, however, the inflammatory phase does not stop, leading to chronic inflammation. This typically occurs when the foreign material cannot be removed (particularly if it is an implanted material) [2,11]. This is somewhat resolved by the granulation tissue (around the foreign material) turning into circumferential fibrous tissue, “walling off” the foreign material from the rest of the host. The goal of this phase is to form new tissue to fill the defect and re-establish continuity in the tissue. The tissue formed is a mix of granulation tissue and regenerated tissue (dependent on the species and the time to heal). 

There are a number of interrelated bioprocesses that produce the tissue (cells and extracellular matrix [ECM]) as the wound edges move toward the center of the wound until the defect is filled [2,9]. Three of these interrelated events are tissue components that have to migrate inward together: fibroblasts, collagen fibers, and blood vessels. There needs to be a substrate on which the cells and blood vessels can migrate across. The substrate (ECM—mostly collagen fibers) is produced by the fibroblasts if provided with enough oxygen. The fibroblasts need to be within 100 μm of a blood vessel to have enough oxygen to produce the ECM [2,4,10]. The repair phase transitions into the remodeling phase. The closer the repair tissue (provisional matrix) is to the structure and function of the native tissue, the less remodeling needs to occur [2,9,10]. Typically, the more granulation tissue and the more stress on the wound the more wound contraction and scar tissue there will be [2,3,4]. There are a number of underlying pathologies that can alter the healing process or prevent complete healing [2,3,4].

Again, the relative amount of scarring vs. regenerative healing is determined by the time to start and complete healing, which is determined by a number of interrelated bioprocesses including: the amount of inflammation, the length of both the inflammatory phase and the repair phase, the amount of stress on the tissue, and the activity of both macrophages and fibroblasts [2,3,4,11,28,29]. There are many diseases where inflammation leads to scarring and reduction in function including: arteriosclerosis and cirrhosis of the liver [2,3,9].

**Figure 1:**
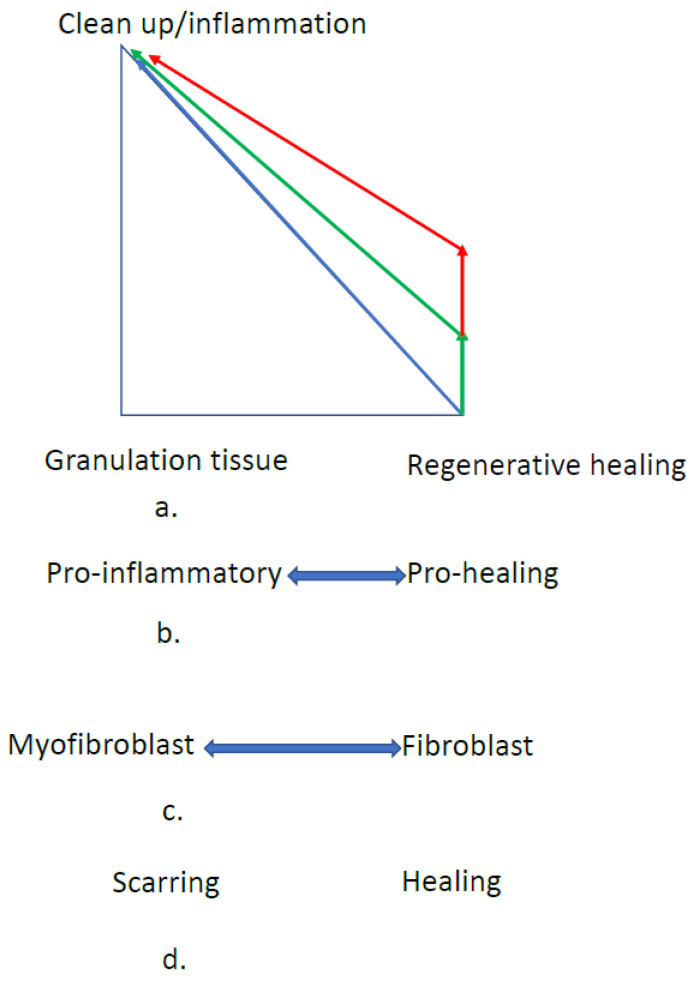
Cell and tissue changes during the repair phase of wound healing. (**a**). As the amount and time of inflammation increases (mostly due to cleaning up of the wound), the higher percentage of healing is granulation tissue. Part of the theory proposed is that animals that are more regenerative would require more clean-up/inflammation before increasing the percentage of granulation tissue formed (green and red lines). (**b**). As the amount and time of inflammation increases (mostly due to cleaning up of the wound), a higher and higher percent of macrophages are pro-inflammatory vs. pro-healing. Again, animals that are more regenerative would probably require more clean-up/inflammation before increasing the percentage of pro-inflammatory macrophages (green and red lines). (**c**). The ratio of pro-inflammatory macrophages to pro-healing macrophages should control the ratio of myofibroblasts to fibroblasts, which in turn will control the ratio of granulation tissue to regenerative healing (**a**), which will control the ratio of scarring to healing (**d**) [14].

Figure 1a shows how granulation vs. regeneration tissue percentages (in the repair phase) as the amount or time of inflammation increases could work [14]. It is likely that even in adults, it takes a minimum amount of inflammation before granulation tissue formation is triggered.

#### 2.5.2. Evolutionary Control of the Adaptive Response

One aspect is the ability to form a blastema. However, epimorphic regeneration is not the goal of healing; it is making the healing more regenerative. In fact, the goal is not to regenerate structure, but to recover function. Although recovery of a specific percentage of a structure is not likely to mean recovery of the same percentage of function, a reduction in scarring will usually mean better function.

Although the evolutionary change is most likely the relative amount of scarring vs. regenerative healing controlled by the time to start and complete healing, it is unlikely to be controlled by a biological clock. As Figure 1 shows, there are a number of bioprocesses that are interrelated. First, the more granulation tissue relative to the regenerative healing, the more wound contraction, and the more relative scarring occurs. Additionally, both the macrophage and fibroblast cells have phenotypes that are more prone to scarring (pro-inflammatory macrophages and myofibroblasts). Also, the genetic change is most likely at the cellular level.

Many authors claim that the macrophage orchestrates the adaptive responses [2,29]. It has a large influence on the immune response by presenting antigens to lymphocytes [2,29]. Since it does control the inflammatory response, it makes sense that it would be the first trigger. There has been a lot of recent work on identifying different phenotypes of macrophages (activity and production levels) [2,14,29].

As seen in Figure 1, the evolutionary change could be the amount of stimulus (e.g., clean-up or stress) required to switch the phenotype to pro-inflammatory. It could be a change in the threshold amount or the slope of the line. As shown in Figure 1, there is probably a relationship between the stimulus and the level of pro-inflammatory macrophages (both the threshold and the slope) [14]. It is, however, unclear if this is a change in type of local macrophages or the recruitment of different types of macrophages. Additionally, it is unclear if they are individual macrophages on a continuum or a relative percent of different phenotypes. What is important, however, is likely to be how this level controls the fibroblast to myofibroblast phenotype switch. Again, the exact relationship is unknown, but increasing the level of pro-inflammatory macrophages would increase the relative amount of myofibroblasts to fibroblasts.

It is also likely that the higher the level of pro-inflammatory macrophages, the longer and more severe the inflammatory process (the observed difference). Then, the transition to the repair phase would involve increasing the level of pro-healing macrophages relative to pro-inflammatory macrophages. Again, lengthening the inflammatory would increase the overall healing time, since little regenerative healing occurs in the inflammatory phase.

Increasing the myofibroblast-to-fibroblast ratio would increase the relative amount of granulation tissue compared to regenerative healing. Then, increasing the relative amount of granulation tissue would increase the relative amount of contraction and scarring. Therefore, the theory is that the evolutionary change is the amount of macrophage stimuli (clean-up or stress) required to shift to the pro-inflammatory phenotype. Again, this could be either a threshold change or a slope change (or both).

To account for the trigger based on the length of time to close the wound, the granulation tissue healing rate could be faster than the regenerative healing rate (or increase over time), leading to a higher relative amount of granulation tissue to regenerative healing over time. It is also likely that the more granulation tissue the faster the contraction rate (making the wound smaller and leading to scar tissue).

Therefore, part of the theory is that the fibroblast-to-myofibroblast ratio set by the macrophage phenotype ratio lasts through most of the repair phase, even though the macrophage phenotype will revert back to pro-healing. This ratio sets the amount of granulation tissue-to-regenerative healing ratio. Therefore, the longer the wound is in the repair phase, the more tissue there is to contract and scar relative to tissue that has regenerative healing.

Again, the theory presented is most likely an oversimplification at best. It will be used to help justify the selection of control strategies. The results of those studies will help show if the theory is useful in selecting control strategies. The goal is not to prove the validity of the theory, but to see whether it is useful in selecting control strategies that could meet the desired outcomes in animal and clinical studies

## 3. Control Strategies

Since the clinical performance requirements as well as the other performance requirements are application-dependent, this section will just give an overview of the types of strategies for controlling the inflammation and repair phases. The emphasis will be on the general strategies vs. how to incorporate biomaterials. The following sections will give more specific examples to show how strategies can be selected and optimized for a few skin injuries, particularly using biomaterials.

### 3.1. Inflammation Control

#### 3.1.1. Causes of Inflammation

Inflammation is the normal adaptive response to help “clean up” the wound after an injury, to prepare the wound for the repair phase. The cells (neutrophils and macrophages) are recruited to remove foreign debris, necrotic tissue and cells, and by-products of hemostasis. These cells not only clean up the wound but also produce cytokines to recruit the cells needed for the repair phase [2,3,4,7,8,29]. These cytokines include ones that are vasodilators (e.g., histamine), which serve to increase the blood flow to the area as well as lead to swelling of the tissue. The increased blood flow will make the tissue redder and warmer, as well as put additional pressure on local nerves causing pain [2,3,4].

The cytokines produced, however, can also have undesirable local and systemic effects [1,3,4,8,29,30]. Locally, they can damage healthy tissue (leading to more inflammation) [4,9]. Systemically, they can increase inflammation in other parts of the body, e.g., many inflammatory diseases including autoimmune diseases and colitis can be made worse or exacerbated by inflammation in other parts of the body or even from foods that increase histamine levels [1,8,9].

#### 3.1.2. Inflammation Control Strategies

These strategies are aimed at reducing the amount of “clean up” needed or the chances of increased inflammation by re-injury or introduction of additional inflammatory materials. In many wounds, the skin is debrided to remove foreign material and necrotic tissue. This serves to reduce the “clean-up” required but also reduces the chance of infection [1,2,3,4,29]. In addition, using coverings that replace the barrier function of the skin can reduce infection, as well as reduce the chance of re-injury. Further, the wound should remain moist to prevent a scab from forming, but not too moist that the exudate can lead to damage to the surrounding skin. Closure of the wound with sutures, staples, glue, etc. can also reduce the chance of re-injury [1,2,4].

However, these foreign materials can also activate macrophages leading to increased inflammatory response. Macrophages can be activated by the size or chemistry of the foreign material [1,3,9,28,29,31,32,33,34]. The chemistry is usually only important if the foreign material is leaching out chemicals or has an antigenic component (normally requiring a foreign protein) [1,3,4].

The size is also important if the foreign material is small enough to be phagocytized [1,2]. If the material is larger than 50 μm the macrophage does not try to phagocytize or surround it, but if it is smaller than 1 μm the foreign material is usually removed easily [31,32,33,34,35]. Fibers, however, can be surrounded by the macrophages but can be too long to be phagocytized [1,3,31,32,33,34,35].

In addition, there are chemical and environmental alterations that can reduce or shorten the inflammatory response. Anti-inflammatory drugs can reduce the activity of cells or the effect the cytokines have on cells. Temperature changes can be used to slow bleeding or increase the efficiency of the inflammatory process. Also, oxygen can alter cellular activity. For example, low oxygen levels (as seen at the beginning of an injury) activate macrophages to produce chemotactic cytokines [1,8,9,36,37,38,39,40,41].

### 3.2. Repair Phase Control

In the skin, the rate at which new tissue fills the defect is controlled by the angiogenic response [1,40,41,42]. The rate of blood vessel ingrowth determines the speed the dermis (connective tissue layer below the epithelial layer) can grow in, and the top layer (the epidermis) requires a well-vascularized dermis to cover the wound [2,12,39,43,44]. Therefore, speeding up the cellular proliferation rate or ECM production rate is only effective if they are slower than the angiogenic rate.

Typically, the healing rate is the speed the epithelial-covered edges move toward the center of the wound. The epidermal migration rate is a good indicator of regenerative healing, while the contraction rate is the speed the wound edges move toward the center of the wound and is more indicative of the amount of scarring [1,2,42]. Therefore, controlling the healing rate is not only controlling the speed of wound closure but also the relative amounts of regenerative healing vs. scarring.

The relative amount of regenerative healing vs. scarring is mostly controlled in the inflammatory phase. However, the quicker the repair phase, the less healing will occur by scarring. Therefore, the main thing to control in the repair phase is the rate of angiogenesis.

To increase angiogenesis, either angiogenic agents or scaffolds or angiogenic agents could be used. The scaffold serves to reduce the need for fibroblasts to produce ECM in order for blood vessels to grow in [2,4]. There are many different types of scaffolds used with a variety of materials, a range of degradation profiles, and different additives (cells, growth factors, etc.) [2,4,30,38,39]. In addition, the angiogenic agents can be used apart from a scaffold delivery system. Environmental factors such as electrical stimulation and hyperbaric oxygen are also used to enhance the angiogenic response [1,2].

A graft has even less of an angiogenic requirement. The blood supply at the wound margins need to attach to the blood supply of the graft for the graft to take [10,43,44]. There are different types of grafts, with different requirements. In addition, the attachment of vessels between the graft and the wound margins can be different in different types of soft tissue. Again, although it normally takes longer for the blood supply to provide enough oxygen for cell survival toward the center of the graft, free skin grafts seem to take, unless the blood vessels become blocked [10,43]. In a number of plastic surgery procedures, pedicle grafts are used which have blood supply from at least one edge and are rotated over the skin defect to reduce the need to have quick attachment between the wound margins and the graft [10].

There have also been many attempts to make graft substitutes work like natural grafts. The blood supply is currently more of a limiting factor for these skin graft substitutes vs. free skin grafts [12,13,14,15,16,17,18,19,20,21]. These graft substitutes can be made by growing them *in vitro* using stem cells (organoids) or with supporting structures (including decellularized tissue or 3D-printed tissue) [2,21,45,46,47].

## 4. Design Principles

### 4.1. General

Although the desired adaptive response(s) is application dependent, the general design principles and strategies to control the adaptive response(s) are relatively application independent. To provide better examples, however, the review will concentrate on open skin wounds, particularly pressure ulcers in the spinal cord injured and full thickness burns.

There are differences in design principles for the three main strategies: use of scaffolds, use of grafts, and treatments without scaffolds or grafts. This section will give some general lessons learned and how they are used to design useful treatments for open skin wounds. This includes how to use clinical performance requirements to help select these designs. Since each application will have different clinical performance requirements, it is important to look at some guiding principles that can help in selecting the clinical performance requirements.

### 4.2. Structure vs. Function

The fields of Regenerative Medicine and Tissue Engineering ascribe to the theory that regeneration of structure is the goal or at least the closer to the native tissue the better. Regenerative Medicine focuses mostly on biological materials—human cells and tissue (stem cells, grafts, transplants, etc.). These biological materials are used to directly replace lost tissue with grafts and/or transplants or use the biological materials to stimulate healing to return to a normal structure.

Tissue Engineering is a broader field in that it uses materials (synthetic and natural) and/or biological response modifiers (growth factors, cytokines and other recombinant products) in combinations not only to replace but also to restore lost tissue and organ functions (biochemical and or structural). The emphasis is on either creating a graft substitute (usually requiring growth in cell culture) or growing the tissue replacement *in vivo*. Therefore, Tissue Engineering not only uses biological materials but also creates systems (*in vitro* or *in vivo*) that serve as graft substitutes or regenerative scaffolds. Again, the emphasis is on recapitulating the native structure with a graft substitute or *in situ* usually with a degradable/regenerative scaffold.

#### 4.2.1. Relationship of Structure to Function

Function is normally the driving force for healing. The injury typically needs to reduce function in some way to stimulate healing [23,24]. This can also happen, without injury, if the load or demand on the tissue or an organ changes [23,24]. In some cases, it is just a discontinuity or a need to remove foreign material. In the skin, the loss of the top layer leads to a decrease in mechanical and barrier function, which becomes the driving force.

Wolff’s law implies biological materials adjust to the applied load [45,46]. Wolff’s law also implies, from a mechanical perspective, that tissue heals better when in a functional situation as long as the load is not too great to cause re-injury [1,2,45,46]. The idea of putting as much load on the healing tissue as possible without re-injury is referred to as Wolff’s Window [1,2,45,46].

Experimental biology has also shown this connection, with regeneration not occurring without a need [11,23,24]. In some cases, this is nerve enervation, in others it is hormonal, and in others it is mechanical. One example is that deer antlers do not reform in castrated animals [11]. This is because the size of the antlers is related to hormones and the ability to reproduce [11]. They can also show male dominance in fighting [11]. One or both of these are needed to attract a mate [11].

Although in most cases recovery of tissue function and regeneration of structure go together, we very rarely are able to recapitulate structure in adult humans [1,2,11]. Again, the typical adaptive response to injury is repair followed by remodeling, which moves the tissue closer to normal [11,23,24].

In most cases, moving closer to the normal structure means increased functional recovery [1,2,23]. The relationship is, however, hardly ever a direct relationship where 50% recovery of structure means 50% recovery of function [1,2,11,23]. In some cases, little functional recovery is achieved until structural recovery is close to 100% [1,2,11,26]. For example, a small imperfection could lead to a weak spot that can lead to mechanical failure at a much lower [1,11]. Similarly, tubular structures can lose barrier function and leak if 100% structural recovery is not achieved [1,23]. It also can be organ functions that require specific cell–cell interactions and/or specific 3D orientation for cells and tissue [1,9].

Although most tissues and organs in the body have structures that have efficient and effective structure-function relationships, that have been guided by evolution and Wolff’s Law development, the key functions can be achieved by other designs. This can be with the use of synthetic biomaterials internally or externally as well as helped with rehabilitation strategies to induce more functional tissue. 

#### 4.2.2. Clinical Performance

From a design perspective, it is function that is important not structure. In rehabilitation, it is functional recovery that is measured not structural recovery [1,2,11]. The purpose of scar tissue is to heal a defect quickly and recover a significant amount of function [11,23,24]. In many cases, there is a trade-off between this speed of healing and the recovery of function [1,2,11,12]. Many disease processes are caused by scarring, either due to the initial healing or a chronic inflammatory response, which allows inflammation and scarring to continue as long as the inflammation lasts [1,2,11].

Therefore, although moving more toward regeneration is a reasonable strategy, the actual goal needs to be functional recovery. For skin wounds, this can also include a cosmetic function, which is more related to structure than other functions. There also can be skin stiffness and recurrence rate; both improved with less scarring.

In general, the normal adaptive response falls short of the desired clinical outcome [6]. It is important to then decide what is/are the desired functional outcome(s), which are currently not being met. Ultimately, there should be a list of desired clinical performance outcomes [6]. This would be the minimum performance level (functional recovery) acceptable. There should also be a maximum level of acceptable risk (harms, complications, etc.) [6]. In most cases, the risks are due to not meeting the clinical performance outcomes. This should lead to an acceptable probability of meeting the clinical performance outcome(s) as well as the acceptable probability of risks [6].

For commercialization of the device, which is needed to have a significant clinical impact, a value proposition has to be proven. This is both on the clinical performance side and on the business side [6]. The risk vs. benefit of the design (performance value proposition[s]) has to be justified as part of FDA approval. Before it is to be commercialized the cost vs. benefit has to also be justified (known as health economics)—both the cost to develop, but also the cost to use. Although in the U.S. this is more about whether the treatment can be reimbursed by insurance, in many countries this is required for the device to be approved.

The use of clinical performance outcomes and risk/benefit analysis is what is lacking in most research studies that are hypothesis-driven (Scientific Method) [6]. It is also the problem with stating the goal of the design is to move closer to regeneration. Proving a design moves closer to regeneration (even a statistically significant improvement) than the current treatment is not a valid reason to select the new design [6]. Besides the need to use functional recovery vs. structural recovery, it is important to determine if it meets a clinical performance requirement not being met by current treatments [6].

Too often, a new device that is proven to enhance regeneration in an animal model, when used clinically, is deemed unsuccessful. This is in large part because the requirement(s) for clinical success was not included in the design process. Again, although the pursuit of graft substitutes grown *in vitro* or made by 3D printing could be feasible someday, we do not currently have the technology to make them meet the clinical performance requirements. In addition, until we can recapitulate structure *in vitro* or *in vivo*, the goal of the skin system should be functional recovery not better regeneration.

### 4.3. Skin Design Constraints

#### 4.3.1. Clinical Performance Design Constraints

Again, the clinical performance design constraints are normally the rate of functional recovery over time, for the minimal acceptable benefit, but would also include the maximum acceptable harm. This may be broken into different constraints such as 50% recovery by 1 month and 90% by 3 months or even broken up by function. Although they are different for different applications, they again are usually related to healing (recovery) time and amount of scarring, which are interrelated. The performance requirements of the system/treatment are the healing rate and quality (scarring vs. regeneration) necessary to meet the clinical performance requirements. Different approaches typically need different bioprocess rates to meet the system performance requirements, which are necessary to meet the clinical performance requirements. Healing time is mostly recovery of barrier functions, which do not need to be provided by wound dressings anymore. It also usually is the point when rehabilitation treatments can start. Limiting scarring can be either a functional or cosmetic requirement. Scarring can lead to too much stiffness, which can reduce movement. Scarring in meshed grafts can lead to a mesh-like appearance of the skin long after the skin graft heals.

#### 4.3.2. Approaches to Meet the Performance Design Constraints

The purpose of this section is to begin the design process by articulating the desired clinical performance for three different scenarios. Skin grafts and skin substitutes work similarly and “heal in” versus a scaffold, which stimulates the tissue to grow in and eventually replace it.

##### Skin Grafts

Although there is not a measurable healing rate, the percentage of the graft that takes or heals-in determines the functional recovery. The rate of revascularization determines the graft’s take. Again, wound preparation and graft thickness are important for a good functional recovery. It does eventually get remodeled so it acts more like a provisional matrix than a graft [2,10]. Although skin grafts are not synthetic biomaterials, using biomaterial tissue adhesives can improve the rate of revascularization.

##### Skin Graft Substitutes

These are designed to work like grafts and therefore require quick revascularization to heal-in. Although progress has been made, it has not been possible to cause the blood flow to be re-established as quickly as with skin grafts and therefore they act more like scaffolds. However, they do not have the pore size and structure to serve as good scaffolds, plus there are areas of dead tissue, which have to be replaced [2,10]. This is a good example of why closer to recapitulation of the normal structure (graft substitute to normal skin) can lead to a poor clinical result, since we cannot currently make “graft substitutes” that work like skin grafts [2,10]. Even with cell seeding the grafts work more like drug delivery systems from the cells vs. the organoid formation desired [1,2,10]. It is likely that someday we will be able to design a graft substitute that can revascularize close to the same time frame as actual grafts; however, with current technology, we are not able to meet the clinical performance design constraints [1,2,10].

##### Scaffold Systems

The main use of biomaterials in skin wounds is for scaffolds. Scaffolds work by tissue growing into them and are typically degradable. Therefore rates of cell, tissue, and vascular ingrowth are the key bioprocesses. These are affected by degradation rate as well as drug delivery rate provided by seeded cells or other drug delivery systems [1]. Different systems will have different performance requirements for these bioprocesses to achieve the desired healing rate. To help illustrate this, a specific example will be used as well as be explored in more depth later.

In this case, pressure ulcers will be used and the clinical performance requirement is to heal the wounds using a scaffold system to make the functional recovery time comparable to surgery (normally a pedicle rotation flap), which is about 6 weeks. The increase in healing rate needed will be explored for a few scenarios [48]:

1. Epithelialization rate (ER) and contraction rate (CR) occur independently of tissue fill rate (TF_rate_), as is often the case with shallower wounds;
2. ER only occurs as fast as the tissue fill rate with CR probably dependent on TF_rate_, as is often seen in deeper wounds;
3. Undermining and other factors require that wounds have higher percentages of tissue fill before significant epithelialization and contraction can occur.

For simplicity, wounds that are cylindrical in nature will be modeled. In scenario 1, TF_rate_ can be neglected. In scenario 2, ER is equivalent to TF_rate_. In scenario 3, a certain amount of TF needs to occur before there is any ER or CR. Based on previous animal studies, for scenario 3, a tissue fill of 80% was selected to be required before ER or CR could begin. In all cases, the overall healing rate is determined by the sum of ER and CR. In some cases, the relative amount of regenerative healing (approximated by ER) vs. scarring (approximated by CR) may be part of the design constraints.

**Figure 2:**
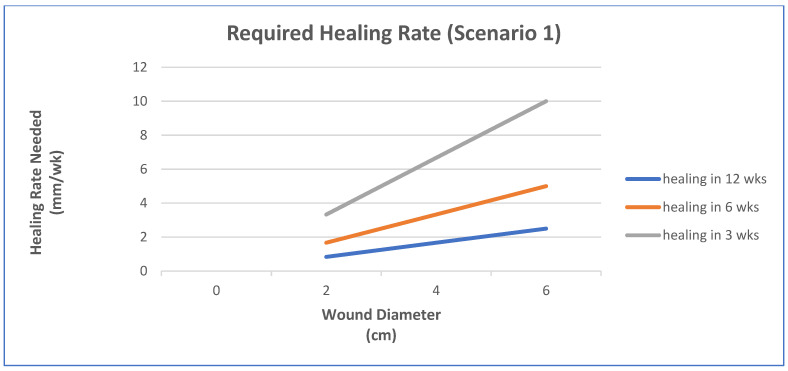
Graph representing healing rate needed if healing is to occur within the specified time for the given wound diameter. This represents scenario 1.

**Figure 3:**
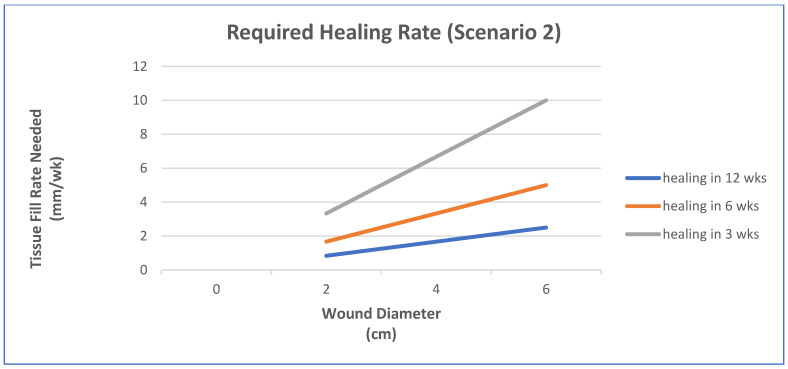
Graph representing the tissue fill rate needed if healing is to occur within the specified time for the given wound diameter. This represents scenario 2.

**Figure 4:**
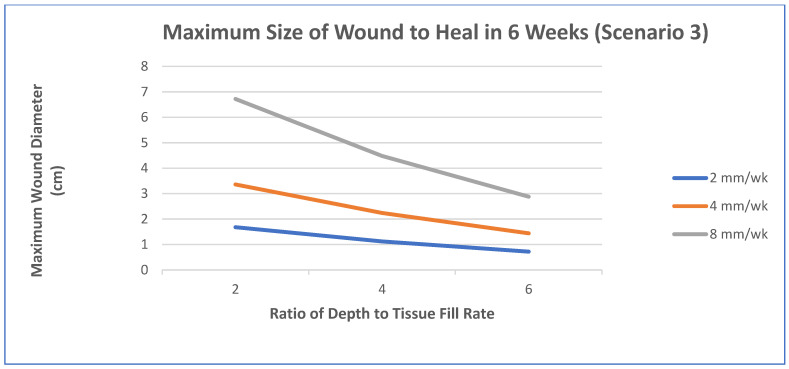
Graph illustrating the maximum size of wound that could heal in 6 weeks with 3 healing rates for a given depth to tissue fill rate (assuming healing does not start until 80% of the tissue fill is complete) (Scenario 3).

The three figures (Figure 2, Figure 3 and Figure 4) show the influence of various parameters such as wound diameter and wound depth on the healing rate for each of the three scenarios. In shallow wounds (Figure 2), the healing rate needed is directly proportional to the wound diameter [48]. For deeper wounds, as in scenario 2 (Figure 3), the healing rate is still proportional to diameter, but the ER is tied to the TF_rate_ [48]. In scenario 3 (Figure 4), there is a lag period that is dependent on the ratio of wound depth to TF_rate_, which essentially just lengthens the healing time [48]. For example, with a wound depth five times that of the TF_rate_, 80% fill will occur in 4 weeks, and the maximum wound size that can heal in 6 weeks at an HR of 4 mm/wk is 1.4 cm [48].

This is just one example of a way to use healing models to obtain approximate performance design constraints for a system, in a specific wound, to meet or exceed the clinical performance requirements. In many cases, not all the rates are known and have to be determined experimentally. It may also require multiple studies to obtain a more accurate model. The real benefit will be discussed later when the selection of strategies is discussed. It will help dictate which combinations of strategies are required to obtain the desired healing rate to meet the clinical performance requirements or if it is even feasible to meet them for a specific case. Again, you ultimately want to know if the health economics and other risk/benefit concerns indicate that the treatment benefits are worth the costs to develop and use. It is therefore likely that for some wounds (particularly larger ones) the benefits may not be worth the added costs.

## 5. Controlling the Adaptive Response to Skin Injury

This section will give more specific strategies to alter and therefore control the adaptive response to injury. The next section will explain how they can be used with scaffold systems (biomaterials) with two specific examples covered in the following section.

### 5.1. General Strategies

In the skin, the normal adaptive response is repair, which goes through three phases: inflammation, repair, and remodeling [1,2,10]. The goal is to control this adaptive response to achieve the desired clinical outcome. Although the desired clinical outcomes are application dependent, it again can usually be expressed in terms of functional recovery; both amount and rate of recovery. Achieving this outcome is controlled by the two interrelated bioprocesses of healing rate and the amount of scarring [1,2,10]. These two bioprocesses can be altered by controlling the three phases of the normal adaptive response to injury.

This section will look at strategies to control the first two phases of the normal adaptive response: inflammation and repair. For most of these strategies, specific research studies will be cited to illustrate how changes in design can be used to control the adaptive response.

### 5.2. Inflammation Control

#### 5.2.1. Relationship to Clinical Outcome

Again, both the level of inflammation and the time to resolution of the inflammatory phase can alter the adaptive response. The level of inflammation can directly affect the amount of scarring. In many cases, the level of inflammation also alters the length of time to resolve the inflammatory phase or is indicative of a chronic inflammatory response. The inflammatory response normally (in adults) needs to resolve before the repair phase can begin. Lengthening the healing time reduces functional recovery by increasing scarring (due mostly to slowing the recovery rate).

#### 5.2.2. Limiting the Inflammatory Response

This can be reducing the amount of material to clean up or increasing the efficiency of the clean up. It also can be achieved by preventing the response from being extended and turning into a chronic response.

The amount of material to clean up can be reduced by debriding the wound, to remove foreign material and necrotic tissue. In addition, stopping the bleeding reduces fibrin build-up. Suturing of an incision wound helps to reduce the amount of fibrin build-up and amount of scarring. Additionally, fibrin and other tissue adhesives can be used as sealants to stop the bleeding or to attach the edges of the wound together like sutures [49,50]. Although fibrin can increase scarring in an incisional wound, it can serve as a scaffold and prevent shearing forces from breaking newly forming tissue [50].

Increasing the number of macrophages and their efficiency in cleaning up the wound can shorten the inflammatory period [1]. Oxygen levels and electrical stimulation have both been used to shorten the time to clean up the wound [1,2]. Low oxygen (below the level in the air) helps to activate the macrophages to both clean up the wound as well as produce cytokines to attract cells to help in the repair process [1,2,29,38,39,40]. High levels of oxygen can help in the healing process but can reduce macrophage activity [37,38,39,40]. It normally is best to use an occlusive dressing (low oxygen permeability) and/or have the higher levels of oxygen be transported by the blood (by inspiring oxygen) to create an oxygen gradient, with macrophages in the center of the wound having low oxygen levels [1,2,37,38,39,40]. Electrical stimulation can do different things depending on how it is used but can shorten the inflammatory response in part due to recruiting macrophages [1,2]. The body uses heat, as part of the inflammatory response, to help make the clean-up more efficient [1].

There are a number of things that can extend the inflammatory phase including: damaging newly formed tissue, reaction to a foreign material, or an infection [1,4]. This can turn an acute inflammation into a chronic one. These were mentioned before and serve more as a list of things to do or avoid.

Wound preparation and care are important to reduce the amount of material to clean up as well as to reduce the likelihood of infection [1,2,4]. Stabilization of the wound to prevent microtears is also important [1,2,4,10]. If foreign materials are used they should be selected to limit macrophage activation [1,4]. This would be to not leach out chemicals that directly or indirectly activate macrophages or be in the 1–50 μm size range [1,2,4].

Infection prevention and treatment normally use systemic antibiotics. However, biomaterials can be used to release antimicrobial agents (antibiotics or metals such as silver and copper) or have antifouling surfaces to prevent bacterial attachment [1,2,43,51,52,53,54]. The problem is biofilms once formed on a biomaterial are difficult to remove and protect the bacteria from antibiotics, usually necessitating the removal of the implant [1,2,43]. Therefore, with biomaterials, it is important to prevent a biofilm from forming.

Further, forming a tight seal at or just below the epidermis can prevent bacteria from getting into the tissue [52,53,54]. This is what the periodontal ligament does for teeth and was used as a strategy for devices, such as catheters, that penetrate the skin for more than a week [46].

Again, inflammation and scar formation is the normal adaptive response to remove or wall off the foreign material. Besides the previously mentioned concerns, there are other things that can increase or decrease the inflammatory response. First, there are drugs designed to reduce inflammation by a number of different processes. There are also a few things that can be changed in the surface of the biomaterial that can alter the inflammatory response including texturing and stiffness [1,4]. Texturing has been used to decrease the thickness of the fibrous capsule formed [4,12]. Surface stiffness was also shown to alter cell activation [1,4]. Additionally, the presence of uni-axial tensile loading was shown to increase scar formation [1,2,4].

### 5.3. Repair Control

#### Relationship to Clinical Outcome

**Figure 5:**
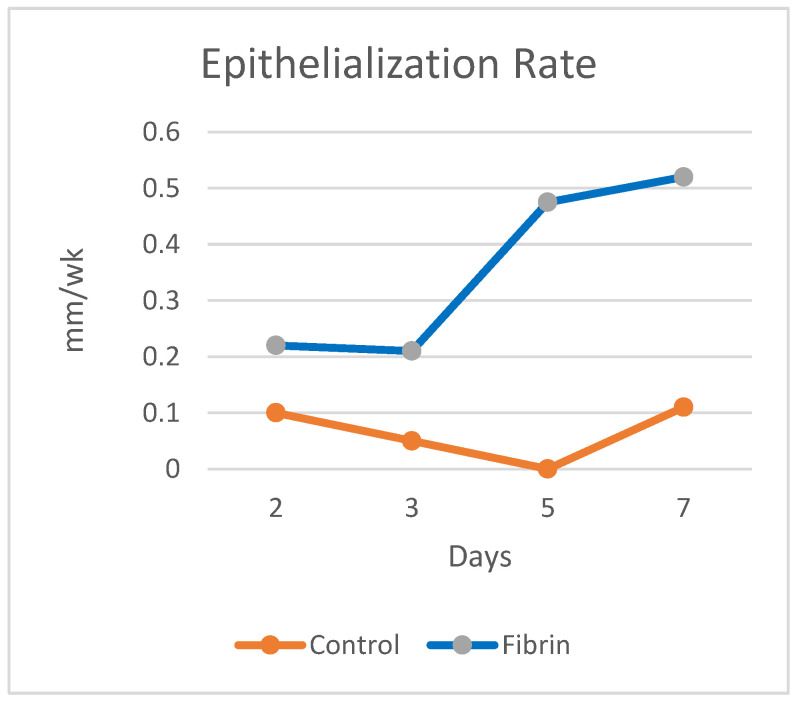
The average epithelialization rate (cm/wk) of the fibrin-treated meshed grafts and controls for the first seven days [56].

The length of time to finish the repair phase is the most critical bioprocess to affect functional recovery and the amount of scarring [1]. The delay in the repair phase due to inflammation and the speed of healing in the repair phase determine when the repair phase is complete. Therefore, control of the inflammatory process is an important part of the control of the repair phase. In a burn study, fibrin used for hemostasis and better graft attachment to the wound bed led to the beginning of the repair phase five days earlier (Figure 5), completion of the repair phase earlier, and a better clinical outcome [55,56].

The speed at which new tissue fills the defect space determines the length of time it takes to repair the defect. The rate of angiogenesis controls the rate of tissue ingrowth [38,39,40,41,42]. However, the blood vessels need a scaffold to grow into the defect. The scaffold used can be tissue ingrowth or an implanted scaffold [1,2]. The next section will be divided into two parts: the use of scaffolds and system design. The emphasis will be on how to increase tissue ingrowth rate to increase healing rate to improve clinical outcomes (by reducing wound contracture and therefore scarring).

## 6. Skin Scaffolds

### 6.1. Variables to Control

In order to design a skin scaffold to meet the functional clinical performance requirements, it needs to be a degradable/regenerative scaffold [1,2]. Although the performance design constraints are different for different applications, the variables that can be modified are the same. Clinically, due to regulatory costs and health economics (costs vs. benefits), the useable designs cannot include all the strategies described. The intent is to present different strategies with some results *in vivo* and clinically. The goal is to first describe what variables can be altered to control the adaptive response with an indication of what is possible. Then, to see how these strategies can be used to design systems for two applications: pressure ulcers and burns. The strategies will be divided into general bioactivity variables and scaffold material variables. General bioactivity variables include environmental changes such as oxygen gradients and electromagnetic fields. Scaffold materials variables include the type of material, degradation rate, configuration, and drug delivery kinetics.

The ability to control the adaptive response with changes in these variables will be explored. Changing the variables can affect bioprocesses at the cellular level, which affects bioprocesses at the tissue level, which controls the tissue ingrowth rate, which controls the healing rate, which controls the functional recovery [1].

#### 6.1.1. Bioactivity

##### Cell Migration

To design a useful system, it is important to know the migration rate limits of the two critical cells: fibroblasts and epidermal cells (keratinocytes). Fibroblasts can migrate up to 200 µm/day [20,21]. Epidermal cells can migrate at 0.33 mm/week over a vascularized dermis. It is only half that amount, if it has to burrow under necrotic tissue to find a good blood supply. For both cell types, there needs to be a substrate that the cells can migrate on. For tissue ingrowth into a porous implant, with no scaffold system, the fibroblasts will produce the ECM. However, fibroblasts will only produce ECM if the blood vessels and nutrients are within 100 µm [2,40]. Therefore the angiogenesis rate controls both the epidermal and fibroblast migration rates.

##### Angiogenesis

Blood vessels can connect through a 1 mm thick collagen/GAG system within 7–9 days [20,21]. This is 50–70 µm/day or 0.35—0.5 mm/week, which is comparable to the epidermal rate, but less than one-third of the fibroblast migration rate. Although the fibroblasts could migrate along a scaffold system, they cannot be more than 100 μm ahead of the blood vessels to produce the ECM scaffold and adventitia necessary for the blood vessels to grow in. The epidermal cells need not only blood vessels to grow in, but a vascular bed to grow over, which would take at least a week for the 1 mm collagen/GAG template) [1,2,20,21].

Angiogenesis is also important for preventing infection in the scaffold by bringing the cells to kill bacteria [1,2,43,44,57]. Angiogenesis is not as important if growing the scaffold *in vitro* since nutrients and antibiotics can be directly administered [5].

Since angiogenesis is the rate-limiting step, speeding it up is necessary to speed up tissue ingrowth. Specific strategies include the use of angiogenic growth factors such as fibroblast growth factor FGF [1,2,27]. Modifying the configuration of the implant is also a good strategy including limiting the thickness of the matrix to less than 200 µm as well as optimizing the pore size and shape to serve as a good scaffold [1,2,53]. Further, cells that induce blood vessel formation (vasculogenic cells, e.g., endothelial progenitor cells) can be seeded in the implant or injected around the implant as well as systemically (and home to the wound) [58].

##### Drug Delivery

Chemical biological response modifiers (e.g., growth factor or cytokine) can be delivered to the wound site to enhance healing. Again, in most cases, the angiogenic (or vasculogenic) response has to be increased before any cellular migration rate can be increased. Natural biomaterials (including fibrin and albumin) can deliver these factors in many ways [49]. One useful way is when they are incorporated into the biomaterial and are only released when the material degrades enzymatically [49,59,60].

The release is controlled by phagocytic cell infiltration related to the tissue ingrowth rate and therefore under biofeedback control. Additionally, this type of release leads to the appropriate gradient to stimulate angiogenesis and tissue healing [49]. Most of the chemical biological response modifiers (particularly angiogenic growth factors) have a short half-life *in vivo* making them difficult to use effectively and efficiently in clinical studies [45]. Natural polymers (such as albumin and fibrin) can serve to protect the biological response modifier from breakdown prior to release [49]. These angiogenic growth factors are only needed if the clinical performance design constraints cannot be met without them, since they increase the difficulty of getting FDA approval as well as increase development and manufacturing costs [1,2,30,49,51].

##### Cell Seeding

Cells can be incorporated into the scaffold, injected locally, or systemically [22,49,58,61,62,63,64,65,66,67,68,69]. For the skin, normal resident cells have been used (keratinocytes and fibroblasts) as well as stem cells (mesenchymal stem cells) and progenitor cells (endothelial progenitor cells) [1,2,22,49,58,61,62,63,64,65,66,67,68,69]. In some cases, these cells were modified genetically to over-produce specific biological response modifiers [22].

Typically, the cells are used to enhance regeneration either by angiogenesis or increased activity of resident cells [1,2,49,50]. Because these cells are available locally or home to a wound site normally, the improved functionality of adding these additional cells needs to be worth the added costs. Again, adding any biologics increases the time and cost of the regulatory process as well as the manufacturing process. This essentially means that cells (as well as other biologics) should only be used clinically if they are needed to meet the clinical performance design constraints. Currently, most cells in seeded clinical skin products have a limited lifetime and serve mostly as growth factor delivery systems without getting incorporated into the healed tissue [1,2,58].

Incorporation into a scaffold requires knowing if the viability and functionality are changed due to the production method or time *in vivo*. Angiogenesis again is a key factor: does the blood supply reach the seeded cells (particularly in the middle of the scaffold) before they start dying? It is even unclear how the cells can survive in a free skin graft that takes about a week to have the blood supply connect to the surrounding tissue and be functional [49]. 

The benefit of injecting stem cells locally or systemically has to be determined since again these cells are available *in vivo.* However, our ability to recruit stem cells to a wound decreases as we age and for certain disease states (including diabetes) [1,2,58]. Since only about 10% of stem cells injected systemically arrive at (home to) an injured site, a local injection around the wound could be more efficient [49].

##### Oxygen

Oxygen is another variable that can control the adaptive response. *In vitro*, oxygen at about four times what is normally seen in a wound (160 mmHg) was the level found to cause the most increase in fibroblast activity as well as a decrease in macrophage activity [70,71]. In an *in vivo* study, inspired oxygen at 70% was able to recreate the local 160 mmHg and led to a significant increase in the healing response [72]. If the wound is also exposed to 70% oxygen, it was found that an oxygen impermeable wound cover was necessary to keep the improved healing response in the early stages, but not necessary during the latter stages [72]. In another study [73], a lower oxygen level, which is closer to the more clinically acceptable 6 L per minute, was used and gave a similar response. It appears therefore that in the early stages of wound healing the high oxygen (with an oxygen gradient through the nonvascular parts of the wound) is beneficial for both the fibroblasts and macrophages. However, once granulation tissue is formed (which has a good blood supply) the oxygen gradient is not needed. Further, the apparent optimum oxygen levels can be achieved without the need for a hyperbaric chamber.

##### Electrical Stimulation

Pulsating electromagnetic fields (PEMFs) were also used to control the adaptive response of full-thickness defects in a rabbit model [74,75,76,77]. In one study, a magnetic field of 2–2.8 mT at a frequency of 75 HZ was applied for 4 h every day for one week, which led to a significant improvement in the healing response [74,75]. In a follow-up study, it was shown that different combinations of amplitude and frequency can produce different adaptive healing responses [75,76]. It, therefore, appeared that different amplitudes and frequencies should be used at various stages of healing to optimize the tissue response [75,76,77,78,79,80,81,82].

An *in vitro* study was performed to examine cellular responses to various electrical fields. In this case, three types of skin cells (keratinocytes, fibroblasts, and endothelial cells) were exposed to electrical field gradients over a range of 100–300 mV/mm (similar to the electrical fields seen in vivo during regenerative healing [81,82]) using microarrays and real-time polymerase chain reaction (RT-PCR) to determine the up-regulation of gene expression and production of specific biochemicals (that showed significant alterations in gene expression) [80]. Although the gene expression was altered in 100s of genes, there were only 11 or fewer, for each cell type, that had a significantly changed level of gene expression [78,79,80].

Although the different fields could elicit different levels of gene expression and production of key biochemicals for each cell type, it appears that the normal change in the electrical field during wound healing has a significant effect on the cell activity of key skin cells [77,78,79,80]. In addition, the use of exogenous electric fields, in the same range as observed in typical wounds, could have a significant effect on wound healing as well [77,78,79,80]. Numerous studies have shown the benefits of electrical stimulation, clinically [73,74,75,76,77,78,79,80,81,82,83,84,85,86,87,88,89,90,91,92,93,94,95,96].

##### Examples

Growth factor studies using PDGF, FGF-1, and TGF-β with or without collagen, PLA or fibrin substrates were performed *in vitro* [1,2,97,98,99,100]. Although *in vitro* optimal fibroblast proliferation occurred in the nanogram/mL range, these levels showed no significant effects *in vivo* [97]. This led to *in vivo* studies at higher doses. In one study, TGF-β (2 µg/cm^2^) and FGF-1 (100 µg/cm^2^) were incorporated in a collagen sponge on full-thickness wounds [98,99]. Both showed an increase in angiogenesis, but the release profile of the growth factors was short (about 2 days) and was not much different from adding a topical dose with the collagen implant [98,99,100].

**Figure 6:**
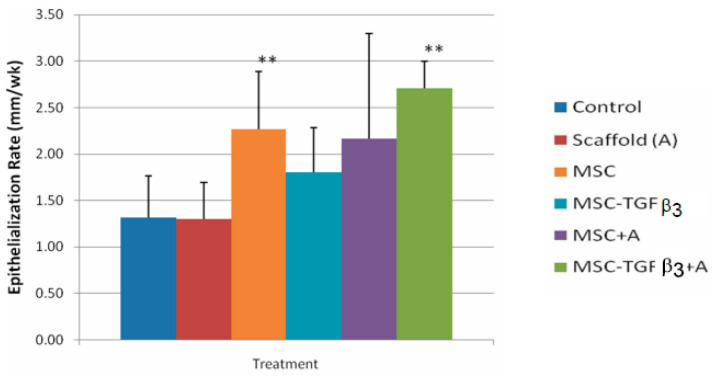
Epithelialization rates at 1 week following surgery. ** indicates statistical significance between treatment and control. ** indicates statistical significance between two treatments (*p* < 0.05) [22].

**Figure 7:**
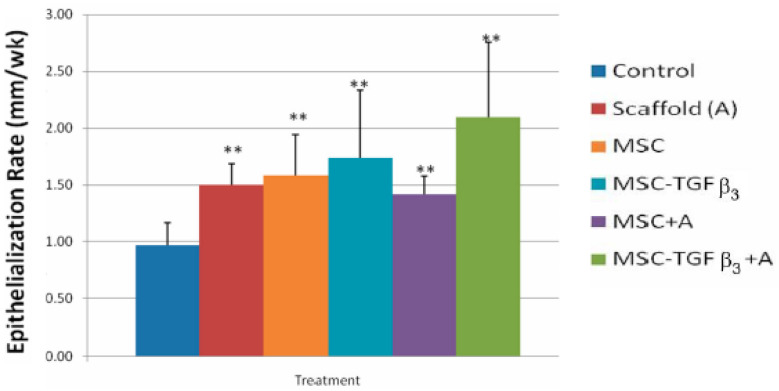
Epithelialization rates two weeks following surgery. ** indicates statistical significance between treatment and control. ** indicates statistical significance between two treatments (*p* < 0.05) [22].

Both mesenchymal stem cells (MSCs) and endothelial progenitor cells (EPCs) were used with and without albumin scaffolds [22,58]. They were injected around the scaffold as well as systemically, in addition to being incorporated into the scaffold to heal full-thickness skin defects [22,58]. The MSCs were also genetically modified to over-produce TGF-β_3_ (a regenerative growth factor) [22]. Using the MSCs incorporated into the scaffold along with local injections led to the biggest effect on the healing rate (doubling of the epithelialization rate at both one and two weeks (Figure 6 and Figure 7)) [15].

Using the EPCs incorporated into the scaffold along with systemic injections produced the biggest effect on healing by the end of the second week [58]. In this study, it (*) appeared that the injected cells did not have a significant effect until after the second week and this was mostly in increasing the angiogenic response inside the scaffold vs. at the scaffold tissue interface [54,58].

#### 6.1.2. Scaffolds

There are many materials that are used as degradable/regenerative scaffolds including: collagen, PLA, fibrin, and albumin [1,2]. Natural tissue adhesives (fibrin and albumin) are a good choice for skin wounds since they can polymerize in the wound conforming to its shape as well as forming a good seal with the wound bed. Additionally, growth factors or cells can be added prior to the polymerization process. Further, the drug delivery kinetics can be tied to the degradation rate, which is controlled by the cellular infiltration rate. This allows the drug delivery kinetics to be controlled by the individual patient’s healing rate [49]. Again, although there are many promising materials and designs for skin scaffolds, the focus will be narrowed to natural tissue adhesives for scaffolds. This is in order to pick systems that were developed to meet the clinical performance requirements for burns and pressure ulcers.

##### Fibrin

Fibrin is produced, *in vivo*, by the polymerization of fibrinogen in the blood. Commercially this sealant/tissue adhesive is supplied with both a human fibrinogen/Factor XIII vial and a bovine thrombin/CaCl_2_ vial. These fibrin sealants were commercially available in Europe starting in 1972. The commercial product was not available in the U.S. until about 2000, requiring the use of autologous or single donor preparations clinically [49,50,101]. The fibrin matrix was used in multiple clinical applications, besides as a tissue adhesive or sealant. It was used as a drug delivery system or cell-seeded matrix for many applications including use as a tissue scaffold [55,101,102,103,104].

Fibrin sealant, when used as a tissue adhesive for skin grafting, has many advantages over using staples including stronger and quicker attachment to the wound bed leading to better graft take and less scarring [55,56,101,102,103,104]. Although it serves as the provisional matrix *in vivo*, making it more porous allows it to serve as a scaffold to increase the angiogenic rate. However, in one study, making it porous significantly reduced the shear strength required to dislodge it from the wound bed and could make it inadequate to handle physiological loading [56,105]. In other studies, it was shown to improve healing in full-thickness defects by primarily increasing the rate of angiogenesis [105,106,107,108,109] as well as could be used for blood vessel anastomosis [56,105,106,107,108,109,110].

The adhesive strength of fibrin (which is near the maximum value within minutes) as a tissue adhesive is proportional to the concentration of fibrinogen prior to polymerization, while the degradation rate is inversely proportional [55,56,103,104,110,111]. Fibrinogen is normally concentrated by cryoprecipitation. However, platelet-rich plasma (which can be made during the surgical procedure) can serve as a tissue adhesive with properties similar to low-concentration fibrin glues [112].

Fibrinogen, however, cannot be treated to inactivate viruses without lipid envelopes such as hepatitis-A and human parvovirus B19 [113]. This is a major reason why it took so long to obtain approval from the FDA [2,49,50]. The clinical product uses donors from a sequestered population, which can be carefully monitored for viruses [113].

##### Albumin

Albumin is a heartier protein than fibrinogen and can be processed at high enough temperatures to inactivate hepatitis-A and human parvovirus [49]. Albumin has been approved by the FDA for a variety of clinical uses [49,113,114,115,116,117,118,119,120]. Although albumin has to be cross-linked (typically with poly[ethylene glycol] (PEG)), it was shown to have as good if not better mechanical properties than commercially available fibrin glues [112,114].

PEG cross-linked albumin was also shown to have better antibiotic properties than fibrin and it can increase the half-life of antibiotics such as gentamicin [50,119,120]. It also exhibits biofeedback-controlled degradation like fibrin. In addition, the degradation rate can be controlled by the type of PEG end groups, which break-down via hydrolysis [101,121].

Albumin glue has comparable wound healing properties to fibrin glue, with no inflammatory reaction observed histologically, although a minimal lymphocytic response is sometimes seen [49]. It does appear, however, that high concentrations of albumin do not degrade as fast as fibrin, leading to a slower increase in wound mechanical properties [49]. Albumin, however, has a number of variables that can be modified to optimize it for a particular application including: the type of functional end-groups on the PEG and the porosity as well as the concentrations of the components used (especially the cross-linking agent and the albumin) [121].

### 6.2. Design Selection

Previous sections have shown typical adaptive responses, why you want to control them, and general strategies of how to control the adaptive response. The review in order to show how the design process can work will become more specific and concentrate on skin wounds. This includes general principles as well as general strategies. This also includes some ways to determine the performance design constraints as well as describing approaches that have a reasonable probability of achieving the clinical performance design constraints.

Along the way, some of the steps in the design process will be described. This section (6.2) will give a more step-by-step approach to the design process. The next section (7) will show examples of parts of the design process for burns and pressure ulcers. As covered before, there are a few key steps needed to design a strategy that are not required in the Scientific Method. These are essentially to define the problem in terms of the clinical performance not currently being met, defining what success looks like, and picking a strategy that has a reasonable probability of being successful (i.e., doing what is needed to solve the problem).

**Figure 8:**
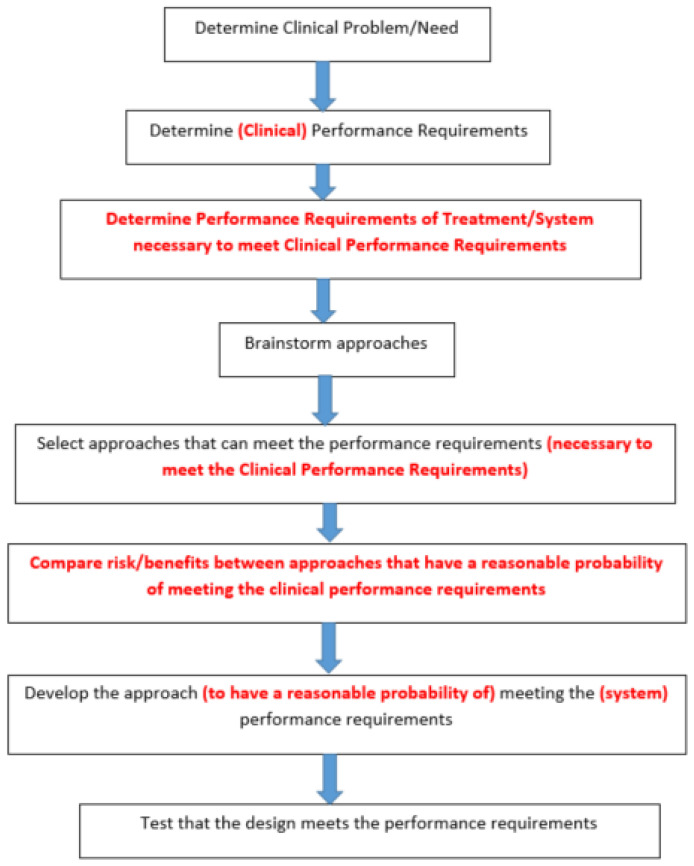
The Engineering Design Process.

Although the engineering design process is normally taught as a linear process, it actually is an iterative process (Figure 8). This is mostly due to the fact that part of the design process is proving that meeting the design constraints at any given level assures meeting the design constraints at the level above it. This is called design verification, which is a big part of getting a device approved by the FDA. Ultimately, the design needs to prove it meets the design constraints all the way up to the clinical performance design constraints (at an acceptable levels), which is called design validation. It is typical that the design constraints have to be modified based on design verification testing. Plus as shown earlier, the performance design constraints of the treatment may be different for different clinical presentations as well as for different approaches. Additionally, most of the design constraints related to business concerns require an iterative process as well.

This Section 6.2, will describe both the linear and iterative design approaches. Again, the following Section 7 will give examples of studies and results used to help design clinical treatment for specific skin wounds.

#### 6.2.1. Engineering Design Process vs. the Scientific Method

Figure 8 (minus the parts in red) shows the linear steps of the engineering design process. The Scientific Method does not require any of these steps until the testing phase. Instead of a problem, all that is needed is a question and a guess of the answer (the hypothesis). The hypothesis is normally written as a statistically significant change that can be noted between treatments, steps in a pathway, etc. Normally it is related to a clinical problem. However, for a comparison of two treatments, all that is required is to prove one treatment is better at something than the current treatment(s), but not whether or not it solves the clinical problem. The engineering design process uses the Scientific Method but has specific requirements that are not typically met in a research study.

These additional requirements are also necessary to justify the need for a research study that is proposing a better solution than what is available currently. Without specifying both what is wrong with current treatments and what success would look like there is no reason to propose a new treatment. There has to be something inadequate about current treatments or there is no reason to look at other options. Additionally, the proposed new treatment should have promise in doing what current treatments cannot or the study is not necessary. The study should then test whether or not the new treatment meets the design constraints of a successful treatment.

It is unlikely that the study will look at all the clinical performance design constraints, but it should be at least one that is currently not being met. The results of the study have to be put in the context of meeting all the clinical performance design constraints, not just the ones shown in the study; suggesting future studies needed to be done. Again, the clinical performance design constraints should include both minimally acceptable benefits as well as maximum levels of acceptable harms (complications).

Once the clinical need is established and the design constraints of a successful device are established, additional design constraints can be included (“would like to” design constraints) such as other desirable, but not necessary, benefits or higher levels of the “have to” benefits as well as lower levels of the “have to” harm design constraints. The next step is brainstorming to come up with potential new approaches. All selected treatments have to have a reasonable probability of meeting the “have to” design constraints. If there is more than one design left, the “best” one would have a desirable mix of the “would like to” constraints or a higher probability of meeting the “have to” design constraints. The selected approach is then fully developed and evaluated to make sure it meets all the design constraints established.

#### 6.2.2. Real World Engineering Design Process for Skin Wounds

##### Real World Design Process

In practice, however, this process is not linear. This also requires separating the design constraints out by level to obtain a design constraint hierarchy. The relationship between levels is the red text added to Figure 8 and the non-linear elements that are described in the next section

At the top of the hierarchy is the clinical performance design constraints, which generally are functional recovery rates (on the business side they are called “value propositions” if they are better than current treatments). On the next level down are the requirements of the system/treatment needed to meet the clinical performance requirements. Typically this is where approaches are brainstormed and selected that have a reasonable probability of meeting the system performance requirements.

It is important to note three truths about design selection that are generally not taught: (1) approaches are usually selected based on meeting the performance requirements of the system/treatment deemed necessary to meet the clinical performance requirements; (2) there is hardly ever a “best” design; and (3) clinical outcomes should include the acceptable probability of meeting the clinical performance (minimum probability of benefits and maximum probability for harms).

The first one is important to make sure approaches are compared with design constraints at the appropriate level. The second and third ones are important since few things are 100% effective or 100% safe and therefore the exact clinical outcome is impossible to predict. You, therefore, select a good approach (one that has a reasonable probability of meeting the clinical performance requirements). The benefit of this is (1) just because, in a particular case, all the clinical design constraints are not met does not mean that the choice was wrong, just that the odds did not work out in your favor and (2) that it reduces the natural instinct to second guess yourself because the outcome of the other choice is a probability as well.

You should further select the approach based on a desirable mix of “would like to” design constraints. The desirability can be determined by a risk/benefit analysis or what are the most favorable trade-offs. The next steps are similar to the steps in 6.2.1. There is, however, likely to be a selection of different designs for the approaches (possible at the whole level and at various component levels) that can be performed with a similar selection process. Again, the key is to select based on design constraints at the appropriate level.

##### Iterative Nature of Design Process

The iterative part of the process mostly comes after testing has begun. Again, the design hierarchy is important here. This is a part of the FDA Design Controls, needed for regulatory approval. Validation tests are to prove that the design meets the clinical performance design constraints. Verification tests are to prove that the system meets the lower level performance requirements (pre-clinical) that enable the system to meet the clinical performance requirements. The pre-clinical constraints are what you believe the design needs to be or do in order to meet the clinical performance design constraints. Most of these pre-clinical ones have to be tested to assure that (1) they allow meeting the next higher-up design constraint all the way up to meeting the clinical performance requirements and (2) the design constraint can be met by the design (verification testing). The iterative nature comes into play when the design does not pass either of these tests.

Again, many of the commercial design constraints require an iterative approach. Although the business design constraints should be taken into consideration in selecting approaches and specific designs, the commercial ability is not really known until the design is validated. Although the clinical performance requirements are the same for all the stakeholders, the commercial ones are not.

In this case, the stakeholders include not only the patient and clinician, but the hospital, insurance companies, investors, stockholders, and company employees. There are trade-offs that have to be made in things such as design, manufacturing, pricing, and company structure in order to meet all the stakeholders’ desires as well as still meet the clinical performance requirements.

## 7. System Design

Again, although there are many promising materials and designs for burns and pressure ulcers, the focus will be narrowed to natural tissue adhesives for scaffolds. This is in order to pick systems that were developed to meet the clinical performance requirements for burns and pressure ulcers. Using a degradable/regenerative tissue adhesive scaffold, three separate systems were developed for two applications: burns and pressure ulcers. For bioactivity, FGF-1 was selected as an angiogenic agent and electrical stimulation was used to mimic the fields in animals that regenerate. Additionally, the use of mesenchymal stem cells and endothelial progenitor cells were explored [22,59].

### 7.1. Burns

A system was developed and tested clinically in burn patients. The performance requirement was to have at least as good a graft take rate as sutures and staples for meshed skin grafts with a significant reduction in stiffness of the healed skin to reduce the rehabilitation time to under 6 months vs. 1–2 years. For the matrix, fibrin was selected to keep the graft firmly attached to the wound bed as well as its ability to deliver and protect FGF-1 [49]. Fibrin had already been used for skin graft attachment in burn patients and it was found that the ability to keep the graft attached to the wound bed improved graft take and reduced scarring [55]. Fibrin was able to speed up the healing rate by shortening both the inflammatory and repair phases [55,56]. From an economic standpoint, it was able to shorten hospital stays, only need minimal post-operative care (no immobilization or pressure dressings), and allow for an earlier start as well as a shorter overall time for physical therapy [55]. This difference, however, needed to be quantified to determine if it would meet the clinical performance requirements.

Previous studies were performed to determine the appropriate method of incorporating the FGF-1 into the fibrin tissue adhesive to obtain the desired release kinetics of the active angiogenic factor [49]. In addition, various changes in the fibrin composition, pore size and porosity were evaluated in animal models in open wounds and for attaching skin grafts [49].

**Figure 9:**
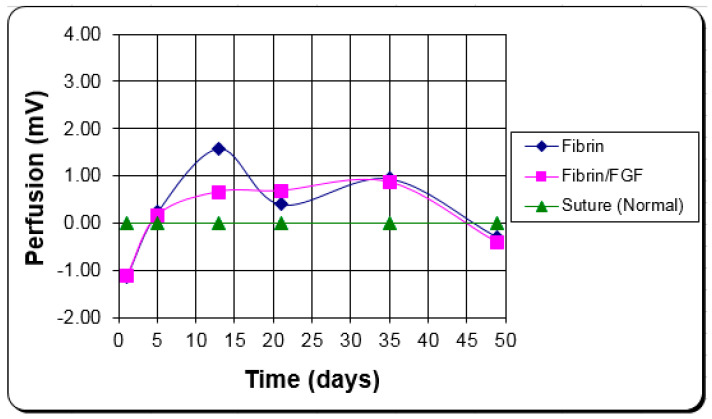
The average perfusion data for the clinical burn patients. The two fibrin treatments are scored relative to the sutured control [56].

**Figure 10:**
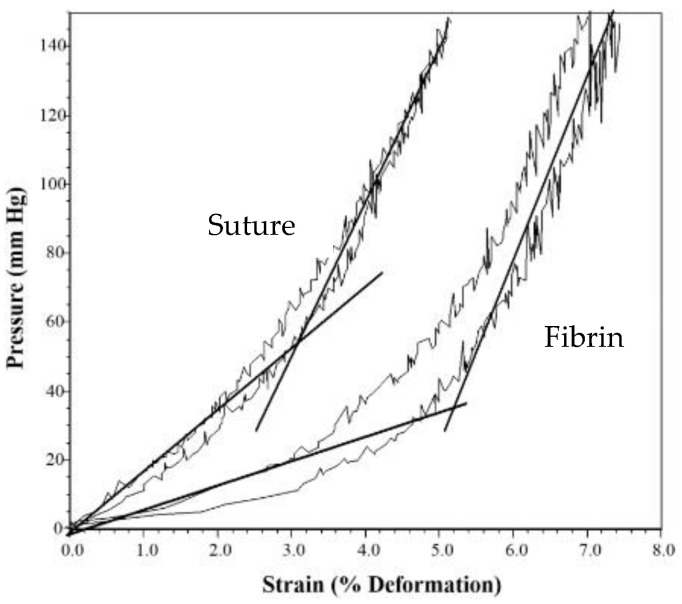
Representative graphs of the stiffness of the treated graft vs. the sutured control [56].

In the clinical study, the fibrin systems were compared side by side to the controls on the same meshed skin graft. The fibrin systems were able to re-epithelialize in two weeks vs. three weeks for the controls by starting the re-epithelialization process about 5 days earlier than the controls (Figure 5) [49,56]. This was attributed to the quicker attachment of blood vessels between the wound bed and the meshed skin graft as well as the ability to keep the blood perfusion up during the entire healing process versus the controls (Figure 9) [56]. Figure 9 shows the blood perfusion (measured in mV with a Scanning Laser Doppler) in the different wound treatments at various time-periods. For each skin graft, the levels above the sutured control given (essentially normalizing the data) were reported. This led to the stiffness of the fibrin system, comparable to normal skin vs. the sutured control, being about twice the stiffness (Figure 10) [56]. The data in Figure 10 were obtained by using a vacuum system placed on the wound and the displacement was measured. Two cycles of off and on were used. The straight lines were to approximate the two regions normally seen in collagen tensile tests where the first slope is the uncoiling of the collagen and the second is the stretching of the collagen fibers. The fibrin systems, therefore, appeared to meet the clinical performance requirements that the sutured control could not.

The clinical results were similar to the previous animal models, although more information was able to be obtained in the animal model. This was helpful in a better quantification of the change in stiffness between treatments. Table 1 shows the elastic modulus of the healed meshed skin grafts after 3 and 10 days. There were six treatments: fibrin without FGF (F), fibrin with FGF (FW), porous fibrin without FGF (PF), porous fibrin with FGF (PFW), sutured control (S), and normal skin (NL). The stiffness was measured in a tensile test on excised wounds of the meshed skin grafts. The ones comparable to Figure 10 (clinical study) are F (fibrin), FW (fibrin with FGF-1), and S (sutured control). Additionally included is NL (normal skin) as well as PF and PFW, which used fibrin that was made porous. The stiffness of the fibrin systems, as in the clinical study, was comparable to normal skin vs. the sutured control (about twice the stiffness) [56].

### 7.2. Pressure Ulcers

Two different types of systems were developed for pressure ulcers. Currently, for (Stage III and IV) SCI (spinal cord injured) pressure ulcer patients, there are two choices: (1) surgical treatment (most commonly skin flap surgery) requiring about 2 months of bed-rest or (2) non-surgical treatment requiring roughly 6 months of bed-rest and continual dressing changes [2]. From a health-economics perspective, it would be beneficial to speed up the non-surgical option to be more comparable to the surgery option without the cost of surgery. In order to approximate the healing time of surgical treatments, a non-surgical treatment would need to reduce healing time by about half (i.e., the performance requirement is to double the normal healing rate) [2].

#### 7.2.1. Electrical Stimulation

Although not a biomaterial solution, the goal is to eventually use it with a scaffold system. However, since the scaffold systems were not clinically approved yet, it was designed to be used without a scaffold first.

In the 1960s, Becker [122] developed the concept of “current of injury”, in which an injury causes a localized shift in current flow. In animals that regenerate, the current of injury is different than in ones that scar [2]. In fetal development, changes in the electric field can lead to changes in structure, causing appendages to grow [2].

Different types of stimulation were carried out in animal models; both non-contact methods PEMF (pulsatile electromagnetic fields) and direct stimulation [75,76,77,78,79,80,81,82]. For a clinical study, it was decided that the treatment should be easy to use, amenable to a home health environment, and also serve as a wound dressing [82,101]. Additionally, even though different fields do different things, it was decided to pick one type of field.

Based on animal studies, a specific type of direct stimulation was chosen that ended up providing a similar voltage drop to those seen in regenerative animals. Although the PEMF was able to meet the performance criteria by a 75–425% increase in re-epithelialization rate and a 50–80% decrease in contraction rate, the direct stimulation method met the ease of use and ability to serve as a dressing criterion [77,81].

**Figure 11:**
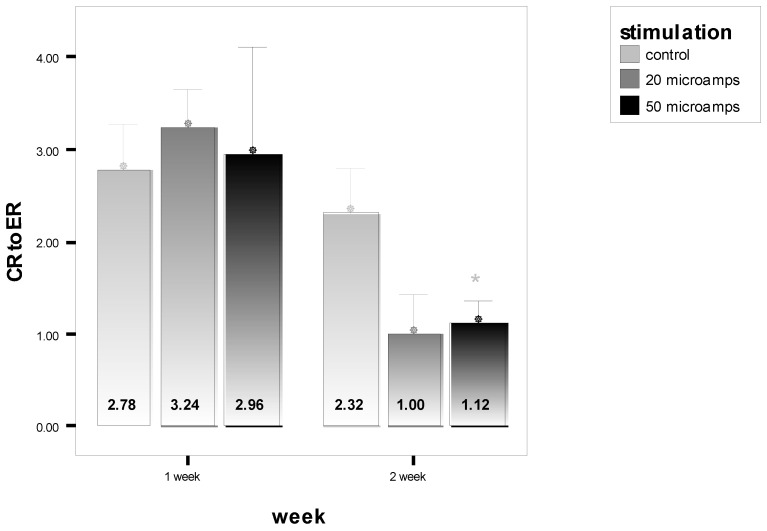
Contraction rate (CR) to epithelialization rate (ER) ratio for the two treatments and the control at 1 and 2 weeks.

In the animal study, with the system designed for clinical use, the re-epithelialization rate doubled between the first and second week and healing was almost exclusively by epithelialization rate (ER) vs. contraction (CR) by the second week. The data shown in Figure 11 [81] have the cumulative CR/ER at both one and two weeks for both a 20 μamp and a 50 μamp electric stimulation bandage compared to a no treatment control.

**Figure 12:**
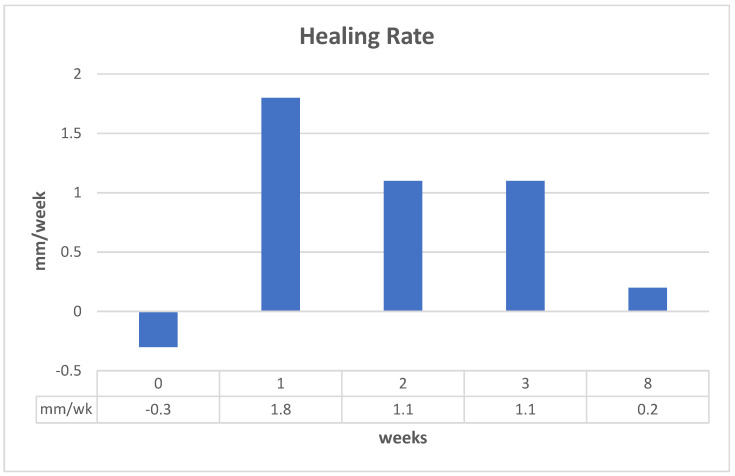
The overall healing rates (mm/wk) of the patients. The values are seen on the left-hand scale as well as included at the bottom of the chart. The values are for the specific time period. Pre-treatment and post-treatment are 0, 1 wk is the first week after treatment began, 2 wk is between 1 and 2 weeks, 3 wk is between weeks 2 and 3, 8 wks are between weeks 3 and 8.

Clinically, the treatment was for eight weeks on and eight weeks off. It, however, was not possible to accurately separate ER from CR so only HR is reported. The data (Figure 12) showing week-to-week healing during the first three weeks, the cumulative eight-week healing, and the non-treated times both before and after the study, showed that the healing rate met the clinical goal for the first three weeks of treatment [81]. In this case, the wounds before treatment and after treatment had little healing. The clinical protocol was then changed to 3 weeks followed by a one-week untreated period [81].

Although it is uncertain why the healing rate dropped off after 3 weeks, since in the animal model, histology was only performed up until 2 weeks, it may be related to lipid membrane disruption. In electroporation, there is a high voltage for a short period of time to disrupt the nuclear lipid membrane to allow the insertion of DNA for recombinant studies [82]. This is generally carried out to cause cells to overproduce a growth factor, similar to studies performed with mesenchymal stem cells [22]. It was suggested that voltage x time is the determinant of membrane disruption [81]. The 20 μamp treatment continuously over a 3-week period has a voltage drop x time close to the voltage time for electroporation [81].

#### 7.2.2. Scaffold Systems

Albumin was selected since studies have shown that the albumin system can perform as well or better than the fibrin systems in terms of strength and tissue response *in vitro* and *in vivo* [50], without the issue of viral contamination. The albumin would be used as a degradable/regenerative tissue adhesive scaffold that would not only conform to the wound but could be delivered in a cheaper home health environment.

Studies were carried out to look at the composition and functional groups on the PEG crosslinker to achieve desirable mechanical properties and degradation profiles [49]. Studies were also performed to look at increasing the vascular response using the angiogenic growth factor FGF-1 used in burns as well as endothelial progenitor cells mixed in with the albumin matrix and injected systemically into the area of healing [49,58]. Although promising, they did not meet the clinical performance goal.

One using mesenchymal stem cells, however, came close [22]. In this case, the mesenchymal cells were treated recombinantly to overproduce TGF-β_3_; a growth factor that is one of the reasons that fetal wound healing is scarless [22]. The cells were used inside the scaffold as well as injected around the scaffold. The treatment, with the albumin scaffold, was able to meet the doubling of the epithelialization at both one and two weeks (Figure 6 and Figure 7) but did not quite get there for HR, since there was also a reduction in contraction rate (62% increase at 1 week and a 40% increase at 2 weeks) [22].

This strategy could be tweaked further but may be sufficient to meet the goal in humans if applied every few weeks since the rabbit wounds contract more than human wounds and the animal model had wounds that would be healed by 3 weeks [22].

## 8. Conclusions

The goal of this review was to explain how to design a biomaterial approach to control the adaptive response to injury, with an emphasis on skin wounds. This included why the adaptive response needs to be controlled, general strategies to control it, and specific strategies related to skin injuries.

The key to the design is meeting the clinical performance design constraints (CPDC)—in this case, restoration of a percent of function within a time-frame (which is dependent on the type of injury). The intent was not to be an exhaustive review of all strategies, with advantages and disadvantages but rather to determine which strategies have a reasonable probability of meeting the CPDC. For a biomaterial, there are strategies related to the structure and ones related to the chemistry (including added bioactivity). The main point of meeting the CPDC is that the requirements are functional, not structural. For skin injuries, although great strides have been made in duplicating skin structure either as a graft substitute or induced using a scaffold, the functional requirements are not met currently therefore these strategies were not described in detail.

As a result, only degradable scaffold systems were explored as a strategy to meet the CPDC. Although there are many potential materials and designs to use, only the fibrin and albumin systems have been evaluated based on CPDC. In addition, the performance requirement of the scaffold system has little to do with the adaptive response to its chemistry, but rather its structure initially and over time. The chemistry, however, is important to improve the scaffolding ability with added bioactivity (cells and biological response modifiers). Another reason albumin and fibrin were selected is that they are tissue adhesives, which do not require a surgical procedure to apply.

The normal adaptive response to a wound falls short of the CPDC. Historically, efforts have been made using various treatments, to control the adaptive response, and these fall into one of three categories: (1) reduce the likelihood of events that could cause additional inflammation (such as infection or re-injury) and slow healing, (2) synthetic materials/devices to restore function while trying to avoid alteration of the normal adaptive response, or (3) using grafts.

Although significant improvements were made in clinical care, in most cases, 1 and 2 have not lead to treatments that met the CPDC. In most cases, not only should the wound be protected during the inflammation phase but efforts should also be made to shorten it. Additionally, there are few devices that do not alter the normal adaptive response and the presence of a foreign material normally leads to a chronic inflammatory response. In addition, the repair phase of healing usually needs to be controlled (sped up) to meet the CPDC.

This has led to more designs that seek to control the repair phase as well as ones that are degradable (or removable) to eliminate long-term chronic inflammation. Although the use of grafts has worked well in many cases, there are many times when they are not sufficient including: lack of donor sites, inability to heal-in (take), and the need to create another wound to heal a wound. This has led many to work on biomaterial solutions, either degradable/regenerative scaffolds or graft substitutes.

A major emphasis of this review is the importance of using the engineering design process to select clinical treatment. Too often clinicians and researchers rely on the Scientific Method for design and assume that statistically better in one area means a better clinical design. A better design has to meet the clinical performance design constraints that are currently not being met (plus all the ones that are) not just be better at something for which the acceptable clinical performance has not been specified.

This thinking was shown in a recent panel discussion at the Society for Biomaterials [123]. The question asked was: “Is it better to regenerate *in vitro* (graft substitute) or *in vivo* (degradable regenerative scaffold)”. Although both sides gave some very good arguments to justify their approach to a particular wound/injury, the question posed misses the point. The CPDC is related to functional recovery rate, not how close to regeneration the end result is. This is particularly important with skin wounds since we are not able to make graft substitutes that work like skin grafts nor are we capable of making degradable/regenerative scaffolds that completely regenerate skin. Although it may be possible someday, until we are able to, the design goal should be to meet the CPDC (functional recovery rate) versus moving closer to regeneration either *in vitro* or *in vivo*.

Another example of this thinking is the notion of 3D printing of skin. Although it seems like a good idea to build up skin layer by layer, with or without cells, to approximate the macrostructure of skin, our resolution currently is not good enough to make a graft substitute that works like a skin graft. In addition, even skin grafts do not become reconnected to the blood supply and resume flow in the first week, which should theoretically be too long for it to take.

One research group claimed that using a bio-ink made of a mixture of extracellular matrix proteins was better than single protein bio-inks since it was closer to being a “graft substitute”. Although it is closer to being like normal skin at the macroscopic level, it does not duplicate the actual arrangement of the individual components of the ECM at the microscopic level.

An important corollary to this, as well as the rest of the review, is that papers or research that claim a better design than currently used need to justify how the study fits into meeting the CPDC, particularly ones that are currently not being met [6,48]. It is still fine to do research on graft substitutes or degradable/regenerative systems, but the results have to be put into the context of meeting the CPDC. Many researchers try to get around this by saying it is applicable to multiple applications, but they still need to justify it for at least one application.

Another aspect of the review is that the engineering design process is iterative in nature, especially if the treatment/system is to be commercialized. It is likely that many studies will show that the performance design hierarchy developed is not accurate or the design cannot meet the design constraint. This will require changes to the design hierarchy, as well as the models used to predict if the performance design constraints can meet the ones a level above. This is also part of justifying the study; not only where the study fits in, but does it re-enforce the CPDC hierarchy or change it?

The important message of the review is that you need to use the engineering design process not the scientific method to design a clinical treatment. This requires establishing a minimum acceptable level of clinical performance. The clinical performance requirements are functional not structural and therefore current attempts to make graft substitutes are not acceptable yet because they cannot achieve functional goals no matter how close they are to recapitulating the native structure. The purpose of this review was not to compare the pros and cons of different strategies as much as how to select strategies that can meet the desired clinical outcome. The last section was to show how well different systems using selected strategies were able to meet the clinical performance requirements *in vivo* and clinically.

## Figures and Tables

**Table 1:** Elastic modulus (PSI).

		3 Day		10 Day
**F**	N = 5	63.0 *±* 28.5	N = 5	57.4 *±* 43.9
**FW**	N = 5	34.4 *±* 15.7 *	N = 5	51.6 *±* 29.6
**PF**	N = 4	46.34 *±* 32.1	N = 5	30.5 *±* 21.6
**PFW**	N = 5	54.1 *±* 32.1	N = 5	54.4 *±* 35.2
**S**	N = 5	101.5 *±* 41.8 *	N = 5	123.3 *±* 142.5
**NL**	N = 3	56.0 *±* 23.9	N = 5	

Note: superscript text (*) indicates a statistically significant difference between like symbols.
